# Emergence of new leptospiral serovars in American Samoa - ascertainment or ecological change?

**DOI:** 10.1186/1471-2334-12-19

**Published:** 2012-01-25

**Authors:** Colleen L Lau, Chris Skelly, Lee D Smythe, Scott B Craig, Philip Weinstein

**Affiliations:** 1 School of Population Health, The University of Queensland, Herston, Queensland, 4006, Australia; 2 WHO/OIE/FAO Collaborating Centre for Reference and Research on Leptospirosis, Queensland Health Forensic and Scientific Services, Coopers Plains, Queensland 4108, Australia; 3 Faculty of Science, Health and Education, University of the Sunshine Coast, Sippy Downs, Queensland 4556, Australia; 4 Barbara Hardy Institute, University of South Australia, Adelaide, South Australia 5001, Australia

## Abstract

**Background:**

Leptospirosis has recently been discussed as an emerging infectious disease in many contexts, including changes in environmental drivers of disease transmission and the emergence of serovars. In this paper, we report the epidemiology of leptospiral serovars from our study of human leptospirosis in American Samoa in 2010, present evidence of recent serovar emergence, and discuss the potential epidemiological and ecological implications of our findings.

**Methods:**

Serovar epidemiology from our leptospirosis seroprevalence study in 2010 was compared to findings from a study in 2004. The variation in geographic distribution of the three most common serovars was explored by mapping sero-positive participants to their place of residence using geographic information systems. The relationship between serovar distribution and ecological zones was examined using geo-referenced data on vegetation type and population distribution.

**Results:**

Human leptospirosis seroprevalence in American Samoa was 15.5% in 2010, with serological evidence that infection was caused by three predominant serovars (Hebdomadis, LT 751, and LT 1163). These serovars differed from those identified in an earlier study in 2004, and were not previously known to occur in American Samoa. In 2010, serovars also differed in geographic distribution, with variations in seroprevalence between islands and different ecological zones within the main island.

**Conclusions:**

Our findings might indicate artefactual emergence (where serovars were long established but previously undetected), but we believe the evidence is more in favour of true emergence (a result of ecological change). Possibilities include changes in interactions between humans and the environment; introduction of serovars through transport of animals; evolution in distribution and/or abundance of animal reservoirs; and environmental changes that favour transmission of particular serovars.

Future research should explore the impact of ecological change on leptospirosis transmission dynamics and serovar emergence, and investigate how such new knowledge might better target environmental monitoring for disease control at a public health level.

## Background

Leptospirosis is the most common bacterial zoonosis in the world, cause by bacteria belonging to the phylum Spirochaetes and genus *Leptospira *[1,2]. Mammals serve as reservoir hosts for leptospires and maintain enzootic transmission cycles within and between animal species. Leptospires have been isolated from almost all mammal species including rodents, livestock, domestic pets, and wildlife. There are over 200 known pathogenic serovars which have host specificity for particular species of animals [2], and host-pathogen adaptation can develop where leptospires colonise the renal tubules of animal hosts without causing any apparent illness in chronically infected animals. When infected animals urinate, the bacteria are excreted into the environment, potentially infecting other animals, and continuing the transmission cycle. Animal species can also be incidental hosts if they are infected with serovars where host-pathogen adaptation has not developed, potentially leading to severe illness or even death. Animal species can be reservoir hosts for some serovars, and incidental hosts for others [3,4]. The presence of particular serovars and their geographic distribution therefore depend on the variety of local animal species, and host-pathogen adaptation of serovars. Emergence of serovars can result from their adaptation to new species of animal hosts, the introduction of infected animal hosts to new areas, or evolution in animal populations and transmission dynamics driven by ecological change.

Humans are incidental hosts for leptospires, and therefore do not contribute to the transmission cycle. Infection can occur through direct contact with infected animals or through exposure to an environment that has been contaminated by animal urine. The usual route of infection is through cuts or abrasions in the skin, but can also occur through intact (especially waterlogged) skin, the conjunctiva, and ingestion or inhalation of water or aerosols. Human infection can result in a wide spectrum of disease ranging from subclinical infections to renal failure, liver failure, pulmonary haemorrhage, and death [3,4].

Leptospirosis is found throughout the world, but is particularly common in tropical and subtropical regions where environmental conditions favour the survival and transmission of leptospires. An estimated 500,000 severe cases occur each year (accounting for only 5 to 15% of all clinical infections), and case-fatality rates of over 30% have been reported in some areas [3,5]. The World Health Organization (WHO) has identified leptospirosis as a neglected tropical disease, and estimates the median global incidence of leptospirosis to be at least 5.1 cases per 100, 000 per year in endemic areas, and 14 cases per 100,000 per year during epidemics. However, incidences vary significantly between regions, with estimated annual incidences per 100,000 per year ranging from 95.5 in Africa, 66.4 in the Western Pacific, 12.5 in the Americas, 4.8 in South-East Asia, to 0.5 in Europe [6].

Most emerging infectious diseases are zoonotic in origin, and ecological changes are major drivers of their emergence [7,8]. Leptospirosis has recently been discussed as an emerging disease in many different contexts around the world [2,9], and different environmental and ecological drivers of disease transmission have been identified [10-12]. Disease outbreaks have been associated with flooding in many areas including India, Philippines, Thailand, New Caledonia, Hawaii, Guyana and Nicaragua [13-19]. Recently, outdoor recreation and ecotourism have emerged as increasingly important risk factors in developed countries [20]. There have also been recent reports on the changing epidemiology of leptospiral serovars and emergence of serovars in the Pacific region [21-24]. In Australia, serovar Arborea was first detected in 1998, and has emerged to become the most common serovar in Queensland, responsible for 35% of cases in 2009 [25]. A recent study in Hawaii also reported the emergence of a new serovar [23].

In American Samoa (AS), the first laboratory confirmed case of leptospirosis was detected in 2003, followed by three additional cases and one death over the next 12 months. In response to this outbreak, the Centers for Disease Control and Prevention (CDC) in Atlanta conducted a seroprevalence study in AS to investigate the epidemiology of leptospirosis and the risk factors for infection. Adults were randomly selected from 13 villages on the main island of Tutuila, and 17% of the 341 participants had serological evidence of previous leptospirosis infection using the Microscopic Agglutination Test (MAT) [3]. On multivariable modelling, male gender and contact with dogs were significant risk factors for being seropositive; and high income and bathing in treated municipal water were associated with being seronegative. The study also made an observational assessment that the risk of human leptospirosis was associated with contamination of streams by rodents, dogs, and particularly pig waste [26].

To further investigate the environmental drivers of leptospirosis in American Samoa, we conducted a more extensive seroprevalence study in 2010, and the methods and results have been described in detail in a recent report [27]. In summary, the study included 807 adult participants from 55 villages on five islands of AS: the main island of Tutuila, the adjacent island of Aunu'u, and the remote Manu'a Islands (Ta'u, Ofu, and Olosega). An overall seroprevalence of 15.5% was detected, with three predominant serovars accounting for over 90% of seropositive tests. Questionnaires were used to assess individual-level risk factors, and geo-referenced environmental data were used to investigate environmental risk factors around the home. Significant risk factors for overall seropositivity included male gender, outdoor occupation, low income, lack of knowledge about leptospirosis, living below the median altitude of one's village, and the density and location of piggeries around the home. Risk factors for infection varied between the three most common serovars: serovar Hebdomadis was associated with outdoor occupations and having piggeries near the home; serovar LT 751 with contact with rain puddles and living further from streams; and serovar LT 1163 with flooding at home, flooding at work, living at lower elevation, swimming, and having piggeries near the home [27].

The present paper adds to this picture by comparing the epidemiology of leptospiral serovars in our study in 2010 with results of the CDC study in 2004; and by examining the association between serovar distribution and ecological zones by using geo-referenced environmental data, including newly available vegetation mapping data [28]. We discuss the serological evidence of serovar emergence in the six years between the two studies, and explore the potential epidemiological and ecological implications of our findings.

## Methods

### Data sources

#### Seroprevalence data

For our seroprevalence study, the microscopic agglutination test (MAT) [22] was used to measure serovar-specific antibodies, and titers of ≥1:50 were considered reactive or seropositive. The study used a MAT panel of 23 serovars, selected based on known epidemiology of leptospiral serovars in the Pacific region, and included serovars that were identified in the CDC study in 2004. Table 1 compares the serovars and serogroups used for MAT in each study. Serovars are divided into serogroups based in their antigenic similarities, and cross-reactions on MAT are known to occur between serovars within the same serogroup [1,2]. Although serogroups are no longer used in the taxonomic classification of serovars, they remain useful for laboratory purposes and epidemiological comparisons.

**Table 1:** Comparison of MAT panels and predominant serovars in 2004 and 2010 leptospirosis studies in American Samoa

		Serovars used in MAT panel		% of total sero-positive tests	
**Serovar**	Serogroup*	**In 2010 Study**	**In 2004 Study**	**In 2010 Study^#^**	**In 2004 Study**

Australis	Australis	✔	✔		

Bratislava	Australis	✔	✔		70.7%

LT 751	Australis	✔		25.5%	

Autumnalis	Autumnalis	✔	✔		

Ballum	Ballum	✔	✔		

Bataviae	Bataviae	✔	✔		

Canicola	Canicola	✔	✔		

Celledoni	Celledoni	✔	✔		

Cynopteri	Cynopteri	✔	✔		

Djasiman	Djasiman	✔	✔		

Grippotyphosa	Grippotyphosa	✔	✔		

Borincana	Hebdomadis		✔		

Hebdomadis	Hebdomadis	✔		48.3%	

Copenhageni	Icterohaemorrhagiae	✔	✔		

Icterohaemorrhagiae	Icterohaemorrhagiae		✔		6.9%

Javanica	Javanica	✔	✔		

Manhao	Manhao	✔	✔		

Georgia	Mini		✔		

Mini	Mini	✔			

Panama	Panama	✔	✔		

Pomona	Pomona	✔	✔		

Alexi	Pyrogenes		✔		

LT 1163	Pyrogenes	✔		17.4%	

Pyrogenes	Pyrogenes	✔	✔		5.2%

Shermani	Santarosai	✔			

Hardjo	Sejroe	✔	✔		

Wolffi	Sejroe		✔		

Tarassovi	Tarassovi	✔	✔		

**Total**				**91.2%**	**82.8%**

*Serogroup designation is no longer used in the official classification and nomenclature of *Leptospiral *serovars, but provided a useful comparison of the two studies.

^#^14 samples were reactive to 2 serovars, and 5 samples were reactive to 3 serovars

Two of the serovars used in our panel are novel, and are in the process of being confirmed and named by the International Committee on Systematic Bacteriology (Subcommittee on the Taxonomy of *Leptospira*) as new serovars:

i. Serovar LT 751 was cultured from urine and kidney samples from rodents during an animal study of leptospirosis in Micronesia in 1995 [29].

ii. Serovar LT 1163 was isolated in 2000 from a traveller who developed acute leptospirosis after a journey to Samoa. No information is available on animal hosts for this serovar.

#### Geographic distribution of seropositive and seronegative participants

Geo-referenced data on island geography and location of houses were obtained from the American Samoa Geographic Information Systems User Group (AS GISUG) [30], and used to geo-locate study participants to their place of residence and map the geographic distribution of seropositive and seronegative cases.

#### Ecological zones

Population density and vegetation type were used as indicators of ecological zones.

Data on point locations of all houses were obtained from AS GISUG, and used to generate a kernel density distribution surface of population density (search radius of 1000 m and grid resolution of 64 m) [27]. Population density (number of houses per square km) at sampled household locations were extracted using GIS, and categorised into three convenience groups of approximately equal numbers (low, medium, and high density) for statistical analysis.

Data from a recently completed vegetation mapping project in AS [28] were used to define vegetation types, and information at sampled household locations was extracted using GIS. For statistical analysis, vegetation types were categorised as urban built-up (impervious urban surfaces such as houses and paved roads); urban cultivated (vegetated areas within a general urban boundary, including fruit trees around homes, gardens, parks, sports fields, and lawns); agricultural (vegetated land used for commercial production); and other vegetation types (including forests, scrubs, marshes, littoral strands, swamps, mangroves, and beaches).

All Geographic Information Systems (GIS) data were collated, stored, mapped and linked using the GIS software, ArcMap v10.0 (Environmental Systems Research Institute, Redlands, CA).

### Statistical analysis

Outcome measures used were seropositivity to any leptospiral serovar, and to each of the three most common serovars individually. Chi-squared tests were used to assess categorical variables and logistic regression was used to calculate the odds ratios of being seropositive.

STATA v11.1 software (StataCorp, College Station, TX) was used, and p values of <0.05 were considered statistically significant.

## Results

### Differences in serovar epidemiology between the 2004 and 2010

Table 1 compares the most common serovars detected in our 2010 study with the results of the CDC in 2004. In our study, the overall seroprevalence was 15.5%, with 125 out of 807 samples reacting to one or more serovars (using a MAT cut-off titre of ≥ 1:50). Fourteen samples reacted to two serovars, and 5 samples reacted to three serovars. Three predominant serovars accounted for 91.2% (136) of the 149 seropositive results. Of the seropositive tests, 48.3% (72) were reactive to serovar Hebdomadis (serogroup Hebdomadis), 25.5% (38) to serovar LT 751 (serogroup Australis), and 17.4% (26) to serovar LT 1163 (serogroup Pyrogenes). Serovar epidemiology differed significantly from findings from 2004 when the overall seroprevalence was 17% (58 seropositive samples out of 341, using a MAT cut-off titre of ≥ 1:100); and 70.7% (41) of sero-positive samples were reactive to serovar Bratislava (serogroup Australis), 6.9% (4) to serovar Icterohaemorrhagiae (serogroup Icterohaemorrhagiae), and 5.2% (3) to serovar Pyrogenes (serogroup Pyrogenes) [26].

### Variation in geographic distribution of serovars in 2010

**Figure 1:**
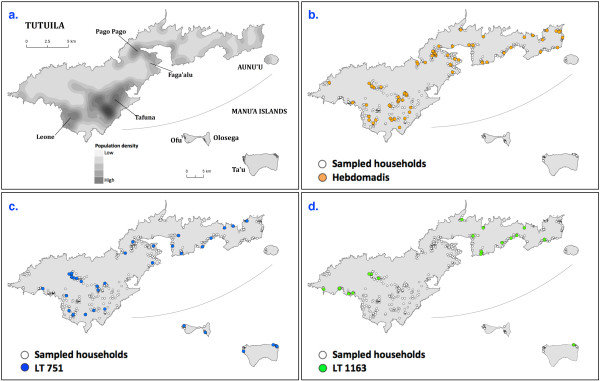
**Distribution of population density and predominant leptospiral serovars in American Samoa 2010**. Panel (**a**): population distribution in American Samoa. Panels (**b**) to (**d**): sampling distribution of the study and positive MATs for (**b**) serovar Hebdomadis, (**c**) serovar LT 751, and (**d**) serovar LT 1163.

Figure 1 shows the population density on the five islands surveyed, the sampling distribution of the 2010 study, and the distribution of seropositive participants for each of the three most common serovars. Panel (a) shows the population density on the islands. Panel (b) shows that seropositive participants for serovar Hebdomadis were distributed throughout Tutuila, but completely absent from the Manu'a Islands. Panel (c) shows that serovar LT 751 was widely distributed on Tutuila and the Manu'a Islands. Panel (d) shows that serovar LT 1163 was completely absent from the most densely populated parts of Tutuila.

### Variation in seroprevalence between islands in 2010

Table 2 shows the variation in seroprevalence between the five islands. Overall seroprevalence varied from 0% on Aunu'u to 16.2% on Tutuila, although there was no statistically significant difference in seroprevalence between the islands. Serovar Hebdomadis was only found on Tutuila, and the seroprevalence of 10% was significantly different to that on the other islands (Chi-squared test, p = 0.002). Serovar LT 751 was significantly more common on the Manu'a Islands (Ta'u, Ofu, and Olosega), and Manu'a residents had an odds ratio for sero-positivity of 2.53 compared to Tutuila residents (p = 0.03, 95% CI 1.07 - 5.98). Serovar LT 1163 was only found on Tutuila and Ta'u, but there was no significant difference in seroprevalence between the islands.

**Table 2:** Leptospirosis seroprevalence in the islands of American Samoa 2010

Number of seropositive samples on MAT and Seroprevalence (%)									
**Island**	**Number Sampled**	**Most common serovars**							**All serovars**
				
			**Hebdomadis**		**LT 751**		**LT 1163**		

	**n**	**n**	**% (95% CI)**	**n**	**% (95% CI)**	**n**	**% (95% CI)**	**n**	**% (95% CI)**

Tutuila	721	72	10 (7.9 - 12.4)	31	4.3 (2.9 - 6.0)	25	3.5 (2.3 - 5.1)	117	16.2 (13.6 - 19.1)

Aunu'u	16	0	0 (0 - 20.6)	0	0 (0 - 20.6)	0	0 (0 - 20.6)	0	0 (0 - 20.6)

Ta'u*	45	0	0 (0 - 7.9)	5	11.1 (3.7 - 24.1)	1	2.2 (0.1 - 11.8)	6	13.3 (5.1 - 26.8)

Ofu*	11	0	0 (0 - 28.5)	1	9.1 (0.2 - 41.3)	0	0 (0 - 28.5)	1	9.1 (0.2 - 41.3)

Olosega*	14	0	0 (0 - 23.2)	1	7.1 (1.8 - 33.9)	0	0 (0 - 23.2)	1	7.1 (1.8 - 33.9)

**Total**	**807**	**72**	**8.9 (7.0 - 11.1)**	**38**	**4.7 (3.4 - 6.4)**	**26**	**3.2 (2.1 - 4.7)**	**125**	**15.5 (13.1 - 18.2)**

*Part of the Manu'a group of islands

### Variation in geographic distribution of serovars in different ecological zones in 2010

Table 3 shows seroprevalences in low, medium, and high population density areas on Tutuila. Comparison of seroprevalences areas using Chi-squared tests showed that the distribution of serovar LT 1163 differed significantly between population density areas (p = 0.001). This serovar was completely absent in areas with high population density, and confirms distribution patterns seen in the maps in Figure 1. There were no statistically significant associations between population density and overall seroprevalence (p = 0.51) or the prevalence of serovars Hebdomadis (p = 0.60) or LT 751 (p = 0.52).

**Table 3:** Leptospiral seroprevalence and population density on Tutuila, American Samoa 2010

Population density	Number Sampled	Seroprevalence of most common serovars			All serovars
			
		Hebdomadis	LT 751	LT 1163	
	**n**	**% (95% CI)**	**% (95% CI)**	**% (95% CI)**	**% (95% CI)**

Low	234	9.0 (5.6 - 13.4) %	5.6 (3.0 - 9.3) %	5.6 (3.0 - 9.3) %	18.4 (13.6 - 23.9) %

Medium	245	9.4 (6.0 - 13.8) %	3.7 (1.7 - 6.9) %	4.9 (2.6 - 8.4) %	15.9 (11.6 - 21.1) %

High	242	11.6 (7.8 - 16.3) %	3.7 (1.7 - 6.9) %	0 (0 - 1.5) %	14.5 (10.3 - 19.5) %

Table 4 shows the seroprevalence in different vegetation zones on Tutuila. Compared to urban built-up areas, residents in agricultural areas were more likely to be seropositive to serovar Hebdomadis (OR 3.04, p = 0.001)) and serovar LT 751 (OR 3.31, p = 0.01). Serovar LT 1163 was more common in 'other' vegetation types that included less populated and forested areas, but this variation was not statistically significant.

**Table 4:** Leptospiral seroprevalence and vegetation type on Tutuila, American Samoa 2010

Vegetation type	Number Sampled	Seroprevalence of most common serovars			All serovars
			
		Hebdomadis	LT 751	LT 1163	
		**% (95% CI)**	**% (95% CI)**	**% (95% CI)**	**% (95% CI)**

Urban built-up	442	7.9 (5.6 - 10.8) %	3.2 (1.7 - 5.3) %	3.8 (2.3 - 6.1) %	14.3 (11.1 - 17.9) %

Urban cultivated	177	10.7 (6.6 - 16.3) %	4.0 (1.6 - 8.0) %	2.8 (1.0 - 6.5) %	16.4 (11.3 - 22.7) %

Agricultural	82	20.7 (12.6 - 31.1) %	9.8 (4.3 - 18.3) %	1.2 (0.0 - 6.6) %	25.6 (16.6 - 36.4) %

Other	20	5.0 (0.1 - 24.9) %	10.0 (1.2 - 31.7) %	10.0 (1.2 - 31.7) %	20.0 (5.7 - 43.7) %

Statistical analyses of ecological zones were only performed for Tutuila because populations on Aunu'u and the Manu'a Islands were too small and localised for meaningful analysis of environmental variables.

## Discussion

This study found that three predominant serovars were responsible for human leptospirosis infection in American Samoa in 2010. To the best of our knowledge, the three serovars were not previously known to occur in the territory. The epidemiology of serovars differed significantly from the CDC study in 2004 with changes in the predominant serovars as well as serogroups. Serovar distribution also varied between islands and ecological zones.

Our findings might indicate true serovar emergence, or an arefactual emergence where the serovars were long established but previously undetected because of limitations in laboratory detection methods. The three most common serovars found in our study were not included in the panel of serovars used for MAT in 2004, and therefore would not have been identified even if they were present in AS at the time. It is difficult to make direct comparisons of predominant serovars between the studies because of differences in the serovars used for MAT, but a comparison of serogroups showed marked differences between the two studies, and indicates that changes in serovar epidemiology were not merely the result of cross-reactions between serovars within a serogroup. Further, the studies also used different cut-off titres for MAT, which makes it difficult to compare seroprevalence, but the predominant serovars remain unchanged if a cut-off titre of 1:100 was used in our study [27]. Sampling distribution also differed between the two studies, with the 2004 study focusing on 13 villages on Tutuila, while our study collected data across Tutuila and four other islands. However, the predominant serovars on Tutuila in 2010 were the same three serovars discussed above (Hebdomadis, LT 751, LT1163), and therefore also differed from those found in the 2004 study. We believe the results provide supportive evidence of true serovar emergence in the 6 years between the studies.

Ecological changes are major drivers of the emergence of zoonotic diseases around the world. Changes in interactions between humans and the environment can result in exposure to different animal hosts; and ecological change can alter pathogen transmission dynamics and consequent disease emergence. For example, leptospiral serovar epidemiology can be affected by change in distribution and/or abundance of animal reservoirs (e.g. pigs); introduction of new serovars through transport of animals (e.g. rats on cargo ships) or natural migration (e.g. bats); or environmental changes (e.g. climate, land use) that differentially favor the survival and/or transmission of specific serovars. Recent reports of changing epidemiology of leptospiral serovars in the Pacific region [21-24] support the hypothesis that ecological factors are likely to be responsible for driving serovar emergence.

Differences in serovar distribution seen in our study could be caused by recent emergence where the pathogens have not yet been introduced to some areas, or the filling of particular ecological niches by the animal hosts of particular serovars. The variation in serovar distribution between islands and ecological zones found in our study might provide insights into possible reasons for the emergence of serovars in AS.

Disparities in serovar distribution between the five islands are likely to be related to variations in environmental conditions. Tutuila is the most urbanized and densely populated island, and home to 95% of the territory's population. The only international port in AS is located on Tutuila, and introduction of animals (e.g. rodents from ships) is most likely to occur here. The Manu'a Islands are very small, remote, isolated, and sparsely populated, with limited air and sea connections to Tutuila. The islands therefore vary in many factors that can affect the transmission dynamics of leptospirosis including animal populations; biodiversity; probability of importation of animals and pathogens (including leptospiral serovars); environmental degradation, pollution, and contamination of streams; flooding risk; and contact between humans and infected animals and/or a contaminated environment. For example, serovar Hebdomadis was only found on Tutuila, and could potentially be a newly introduced serovar from rodents through international shipping.

Differences in geographic distribution of serovars between ecological zones on Tutuila suggest that the three main serovars have different animal hosts that live in distinct environments. Different risk factors for each serovar also support this explanation [27]. For example, serovar LT 1163 is completely absent from high population density areas, and is therefore unlikely to be carried by common peridomestic animals in AS such as pigs, dogs, house mice (*Mus musculus*), Norway rats (*Rattus norvegicus*), or roof or black rats (*Rattus rattus*). More likely animal hosts for serovar LT 1163 include the Polynesian rat (*Rattus exulans*) and the three species of endemic bats, all of which are more abundant in forested areas [31]. In contrast, serovar Hebdomadis was widely distributed across Tutuila and more prevalent in agricultural areas, and is therefore more likely to be carried by one or more of the peridomestic animal species.

Variation in the geographic distribution of serovars in different ecological zones support the hypotheses that environmental factors play an important role in the transmission dynamics of serovars, and ecological change will therefore be a major driver of serovar emergence.

There are limitations to the use of serological methods for leptospirosis, and future studies involving bacterial cultures will be required to confirm the presence of serovars detected on MAT. It is also possible that yet undiscovered serovars in American Samoa might have produced cross-reactions with serovars in our MAT panel. Veterinary studies would provide valuable information on local animal species responsible for the transmission of each serovar, and improve our understanding of pathogen transmission dynamics. Although our study provided supportive evidence of serovar emergence and showed different spatial patterns in serovar distribution, many parts of AS are sparsely populated and a larger follow-up survey would be required to confirm true serovar emergence, determine whether serovar epidemiology is continuing to evolve, and further explore the relationship between environmental factors and serovar distribution.

## Conclusions

Despite limitations in serological diagnosis and possible effects of ascertainment, this paper provides supportive evidence for the emergence of previously unknown leptospiral serovars in AS. Future research should focus on specifically testing the hypothesis that ecological change has direct impact on serovar distribution and disease risk in humans. Such a finding would support environmental monitoring as a valuable tool for identifying high-risk areas for infection and directing public health interventions [32]; and contribute to the growing evidence that ecosystem conservation is beneficial to human health [8,33].

## Abbreviations

AS: American Samoa; AS GISUG: American Samoa geographic information systems user group; CDC: Centers for disease control; GIS: Geographic information systems; MAT: Microscopic agglutination test; WHO: World health organization

## Competing interests

The authors declare that they have no competing interests.

## Authors' contributions

CLL, LDS and PW conceived of the study and participated in the design of the study. CLL conducted the fieldwork, collected all data, and drafted the manuscript. CLL, CS, LDS, SBC and participated in data analysis. All authors were involved in interpretation of results and writing of the final manuscript.

## Authors' information

CLL - MBBS, MPH&TM, FRACGP. PhD Candidate, School of Population Health, The University of Queensland, Brisbane, Australia.

CS - MSc, PhD. School of Population Health, The University of Queensland, Brisbane, Australia.

LDS - BSc. Director, WHO/OIE/FAO Collaborating Centre for Reference and Research on Leptospirosis, Brisbane, Australia.

SBC - MSc, PhD. Supervising scientist, WHO/OIE/FAO Collaborating Centre for Reference and Research on Leptospirosis, Brisbane, Australia. Associate Professor, University of the Sunshine Coast, Sippy Downs, Queensland, Australia.

PW - MBBS, PhD, MAppEpi, MA, FAFPHM. Professor of Ecosystem Health and Dean of Graduate Studies, University of South Australia, Adelaide, Australia.

## Pre-publication history

The pre-publication history for this paper can be accessed here:

http://www.biomedcentral.com/1471-2334/12/19/prepub
